# *Candidatus* Amarolinea and *Candidatus* Microthrix Are Mainly Responsible for Filamentous Bulking in Danish Municipal Wastewater Treatment Plants

**DOI:** 10.3389/fmicb.2020.01214

**Published:** 2020-06-09

**Authors:** Marta Nierychlo, Simon J. McIlroy, Sergey Kucheryavskiy, Chenjing Jiang, Anja S. Ziegler, Zivile Kondrotaite, Mikkel Stokholm-Bjerregaard, Per Halkjær Nielsen

**Affiliations:** ^1^Center for Microbial Communities, Department of Chemistry and Bioscience, Aalborg University, Aalborg, Denmark; ^2^Australian Centre for Ecogenomics, The University of Queensland, Brisbane, QLD, Australia; ^3^Section of Chemical Engineering, Department of Chemistry and Bioscience, Aalborg University, Aalborg, Denmark; ^4^Krüger A/S, Aalborg, Denmark

**Keywords:** filamentous bulking, activated sludge, 16S rRNA amplicon sequencing, *Ca*. Microthrix, *Ca*. Amarolinea

## Abstract

Filamentous bulking is a common serious operational problem leading to deteriorated sludge settling that has long been observed in activated sludge biological wastewater treatment systems. A number of bacterial genera found therein possess filamentous morphology, where some have been shown to be implicated in bulking episodes (e.g., *Ca.* Microthrix), the impact of many others is still not clear. In this study we performed a survey of 17 Danish municipal wastewater treatment plants (WWTPs) with nutrient removal using 16S rRNA amplicon sequencing over a period of 13 years, where all known filamentous bacteria from 30 genera were analyzed. The filamentous community constituted on average 13 ± 6%, and up to 43% of total read abundance with the same genera common to all plants. *Ca.* Microthrix and several genera belonging to phylum Chloroflexi were among the most abundant filamentous bacteria. The effect of filamentous bacteria on sludge settling properties was analyzed using measurements of the diluted sludge volume index (DSVI). Strong positive correlations with DSVI were observed only for *Ca.* Microthrix and *Ca.* Amarolinea, the latter being a novel, recently characterized genus belonging to the phylum Chloroflexi. The bulking potential of other filamentous bacteria was not significant despite their presence in many plants. Low phylogenetic diversity was observed for both *Ca.* Microthrix and *Ca.* Amarolinea, making physiological characterization of individual species and potential development of control strategies more feasible. In this study we show that, despite the high diversity of filamentous phylotypes in Danish WWTPs, only few of them were responsible for severe bulking episodes.

## Introduction

Filamentous bacteria are commonly observed in activated sludge in biological wastewater treatment plants (WWTPs) where they are suggested to importantly provide the structural backbone of well-settling flocs (Seviour and Nielsen, 2010). However, overgrowth of some filamentous bacteria is associated with deteriorated sludge settling, leading to sometimes serious operational problems known as sludge bulking (Liao et al., 2004; Martins et al., 2004; Lou and de los Reyes, 2005; Vervaeren et al., 2005). Some of those possessing hydrophobic cell surfaces can also cause foam on the surface of process and settling tanks. Filamentous bulking and foaming have long been considered as serious problems in biological wastewater treatment systems as they can lead to the carryover of solid particles in the effluent, hence reducing treatment efficiency, as well as having a negative impact on receiving waters. A large fraction of Danish WWTPs with nutrient removal (BNR) experiences temporal bulking problems (Mielczarek et al., 2012) caused by excessive growth of certain filamentous phylotypes. Despite the available knowledge of filamentous bacteria, it is not possible to predict bulking events and the available control strategies are not always effective.

Identification of filamentous bacteria is a crucial first step for successful physiological characterization, and selection of proper control measures. Traditionally, identification of filamentous organisms in activated sludge has been based on microscopic observation of distinct structural features of individual filaments and subsequent classification into a number of different morphotypes (Eikelboom, 1975, 2000; Jenkins et al., 1993). However, it was recently shown that a single morphotype can cover bacteria belonging to phylogenetically distinct groups that likely vary in their metabolic potential (Nielsen et al., 2009a, b; Seviour and Nielsen, 2010). Thus morphotype-based identification underestimates filament diversity, which may obscure the development and application of suitable control strategies.

The development of culture-independent molecular methods has facilitated more reliable identification and description of the diversity of filamentous bacteria present in the activated sludge environment. Fluorescence *in situ* hybridization (FISH) and 16S rRNA amplicon sequencing have been applied to survey microbial communities in activated sludge to identify and characterize filamentous bacteria common in this ecosystem (Mielczarek et al., 2012; Araújo Dos Santos et al., 2015; Milobędzka and Muszyński, 2015; Jiang et al., 2016; Saunders et al., 2016; Wang et al., 2016; Zhang et al., 2019). Filamentous genera found therein span several phyla, i.e., Actinobacteria (Rossetti et al., 2005), Bacteroidetes (Van Veen et al., 1973; Kragelund et al., 2008), Chloroflexi (Speirs et al., 2009; McIlroy et al., 2016), Firmicutes (Liu et al., 2000), Planctomycetes (Liu and Seviour, 2001; Liu et al., 2001) and Proteobacteria (Kragelund et al., 2005, 2006). The filamentous bacteria most frequently linked to operational problems in WWTPs with nutrient removal are the actinobacterial genera *Candidatus* Microthrix (Kristensen et al., 1994; Rossetti et al., 2005; Seviour and Nielsen, 2010), *Gordonia*, and *Skermania* (the latter two sometimes collectively called Mycolata, Carr et al., 2006; Eales et al., 2006; Kragelund et al., 2007a; Seviour and Nielsen, 2010), *Candidatus* Nostocoida (Schade et al., 2002), and several genera belonging to the phylum Chloroflexi (Bjornsson et al., 2002; Kragelund et al., 2007b; Seviour and Nielsen, 2010; Speirs et al., 2011; McIlroy et al., 2016; Nittami et al., 2017). In more simple activated sludge plants with carbon removal and nitrification (often serving specific industries), a number of other genera are commonly encountered including gammaproteobacterial *Thiothrix* and some alphaproteobacterial species, such as *Neomegalonema* (formerly *Meganema*) (Eikelboom and Geurkink, 2002; Kragelund et al., 2005).

Studies of activated sludge microbial community composition can be carried out by 16S rRNA gene amplicon sequencing but the identification of some abundant bacteria has sometimes been hindered by the lack of genus-level taxonomic classification and/or the lack of closely related reference sequences in public databases (McIlroy et al., 2015, 2017). This is notably an issue for the phylum Chloroflexi, which constitutes a significant fraction of the filamentous community in Danish WWTPs (Mielczarek et al., 2012), with most of the abundant phylotypes only classified to the family or order level. Development of ecosystem-specific MiDAS taxonomy containing exclusively high-quality reference sequences from wastewater treatment and anaerobic digesters ecosystem that employs robust taxonomic classification (Dueholm et al., 2019; Nierychlo et al., in press) enables species-level identification of most activated sludge microbes. The combination of high-throughput sequencing technologies with the activated sludge ecosystem-specific database and taxonomy enables detailed profiling of the total WWTP microbiome, allowing the identification of statistical relationships between the abundant phylotypes and process performance parameters.

The aim of this study was to perform 16S rRNA gene amplicon sequencing for the survey of the diversity, abundance, and distribution of known filamentous microorganisms in Danish full-scale WWTPs with biological nutrient removal (BNR), and to investigate the relationship between the abundance of specific filamentous species and sludge settling properties. The comprehensive survey included more than 600 samples from 17 Danish WWTPs collected over a period of 13 years in the frame of the MiDAS project (McIlroy et al., 2015; Nierychlo et al., in press), allowing an unprecedented insight into the dynamics of the filamentous community of full-scale BNR systems.

## Materials and Methods

### WWTPs and Sampling

Biomass sampling and processing were performed for 17 plants in the frame of the “Microbial Database project” (Nielsen et al., 2010; McIlroy et al., 2015). The plants were sampled up to 4 times a year from 2006 to 2018, giving a total of 606 samples. Samples of activated sludge were taken from aeration tank, transported overnight to the laboratory and processed within 24 h. An overview of the plants, i.e., configuration, design, percent of industrial wastes, presence of primary settling and digester as well as number of samples analyzed, is presented in Supplementary Table S1. All plants were municipal WWTPs located in Denmark, treating primarily household wastewater with contributions from industry in the range of 5–70% of the influent chemical oxygen demand (COD). Fourteen plants were designed for enhanced biological phosphorus removal (EBPR), 2 for BNR, and 1 changed from BNR to EBPR during the sampling period. All plants were operated with similar sludge ages of 20–25 during summer and 25–30 days during winter, and all plants had supplementary chemical P-removal and added flocculants intermittently to aid settling. Median temperature was similar in all plants and ranged from 9°C in the winter to 18°C in the summer. pH was stable at 7.2 (± 0.35).

All plants were operated under the effluent quality regulations of 75 mg/L total COD, 8 mg/L total-N, and 0.5–1.5 mg/L total-P. The actual effluent quality was, however, better with median effluent COD, N, and P of 25, 4.1, and 0.3 mg/L, respectively. Diluted sludge volume index (DSVI) was measured on site and provided by plant operators.

### Amplicon Sequencing and Bioinformatic Analysis

Bacterial community composition was investigated using 16S rRNA amplicon sequencing. DNA extraction, amplification of the V1-3 region using the 27F (AGAGTTTGATCCTGGCTCAG) (Lane, 1991) and 534R (ATTACCGCGGCTGCTGG) primers (Muyzer et al., 1993), amplicon sequencing were performed as described in Stokholm-Bjerregaard et al. (2017). V1-3 region was chosen for activated sludge community analysis based on the study of Albertsen et al. (2015) and Dueholm et al. (2019), showing that it covers phylum Chloroflexi phylum better than the V4 region. Forward reads were processed using USEARCH v.11.0.667 (Edgar, 2010) as described by Dueholm et al. (2019) with raw reads trimmed to 250 bp and quality filtered. Exact amplicon sequence variants (ASVs) were generated using -UNOISE3 (Edgar, 2016) with standard settings, and taxonomy was assigned using the MiDAS 3 reference database and the SINTAX classifier with a confidence threshold of 0.8 (Edgar, 2018; Nierychlo et al., in press).

### Data Analysis and Visualization

Data processing was performed using R v.3.5.1 (R Core Team, 2018), RStudio (RStudio Team, 2015). 606 samples with minimum 13,500 reads were normalized to 100% and visualized using ggplot2 v.3.2.1 (Wickham, 2009) and ampvis2 v.2.4.9 (Andersen et al., 2018). Principal coordinate analysis (PCoA) plot with Bray–Curtis dissimilarity was used to visualize beta diversity in the samples. Partial least squares regression (PLS) was performed using R package mdatools v0.10.1 (Kucheryavskiy, 2020) to validate quantitative relationship between DSVI and microbial community composition, and to identify specific microbes which correlate with the DSVI parameter. Only species with median abundance ≥ 0.05 were used to perform the PLS. Systematic cross-validation (venetian blinds) with eight segments was used for model validation. Determination coefficient (*R*^2^) and root mean square error (RMSE) computed for cross-validated predictions were used to evaluate performance of the models. Contribution of each species to prediction of DSVI values was assessed using regression coefficients supplemented with confidence intervals computed using Jack-Knife approach. A detailed time-series dataset with additional 63 samples with higher sampling frequency in years 2012–2014 from Aalborg W WWTP were used for the analysis of the dependence of filaments abundance on sludge settling in that plant; the data was processed as above. Sequencing data is available at the Sequence Read Archive^1^ under the project number PRJNA622675 (general analysis) and PRJNA625645 (Aalborg W time-series). Metadata files and R codes are available at https://github.com/martanierychlo/Filaments_DK.

### Fluorescence *in situ* Hybridization (FISH)

FISH with the NLIMI91 probe targeting the genus *Trichococcus* (Liu and Seviour, 2001) was performed essentially as described previously (Nielsen, 2009) at a hybridization buffer formamide concentration of 20% [v/v] after 30 min lysozyme pretreatment of the biomass at 37°C (concentration of 10 mg/mL). Both the 5′ and 3′ ends of oligonucleotide probe were labeled with the sulfoindocyanine dye Cy3 (DOPE-FISH; Stoecker et al., 2010). The EUBmix probes, targeting most bacteria (Amann et al., 1990; Daims et al., 1999), were labeled with 5(6)-carboxyfluorescein-*N*-hydroxysuccinimide ester (FLUOS). The quantitative FISH (qFISH) biovolume fraction was calculated as a percentage area of the total biovolume, hybridizing the EUBmix probes, that also hybridizes with the NLIMI91 probe. qFISH analyses was performed with the Daime image analysis software (Daims et al., 2006) and each measurement based on 30 fields of view taken at 630 × magnification. Microscopic analysis was performed with a white light laser confocal microscope (Leica TCS SP8 222 X) (Leica Microsystems, Wetzlar, Germany).

## Results

### Composition of the Filamentous Community in Danish WWTPs

**FIGURE 1:**
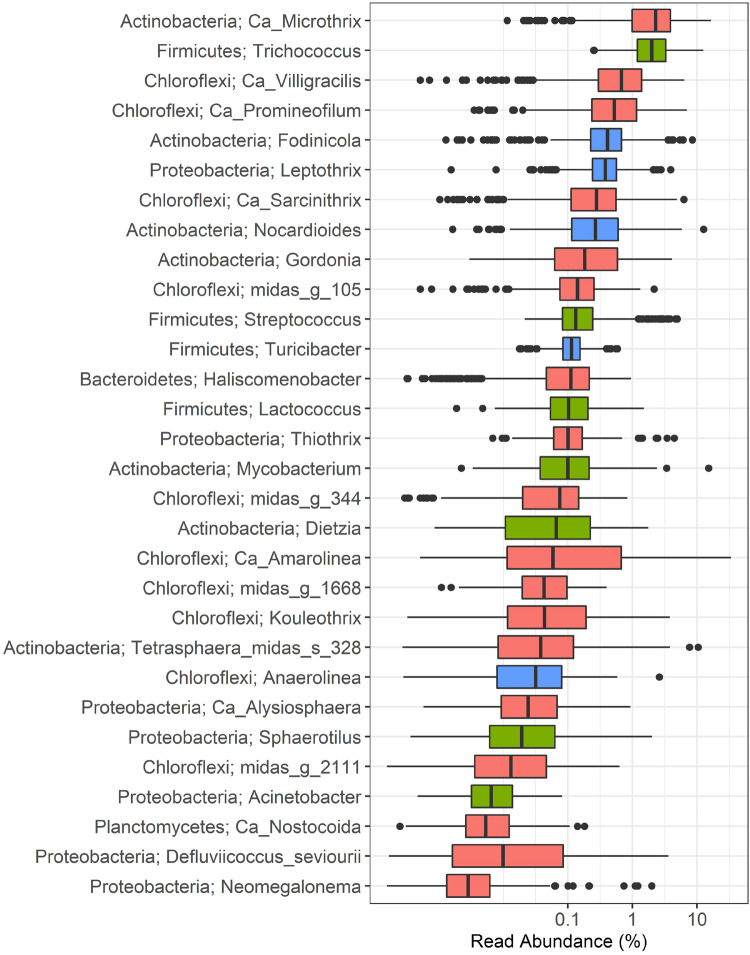
Distribution of filamentous bacteria in Danish municipal BNR WWTPs. Phylum and genus or species names are shown. Each box represents the samples collected from 17 municipal full-scale WWTPs during the 13-year long survey. Coral: filamentous morphology observed *in situ*; green: variable morphology observed *in situ*; blue: filamentous morphology observed in pure culture with no *in situ* information available.

The abundance of filamentous bacteria was surveyed in 17 Danish full-scale municipal BNR WWTPs over a period of 13 years using 16S rRNA gene amplicon sequencing. The genera analyzed represent all phylotypes known to contain species with a filamentous morphology (Table 1 and Figure 1, and Supplementary Figure S1), including genera that are always present in filamentous form in activated sludge (Figure 1, coral); genera with variable morphology in activated sludge (Figure 1, green); and genera, for which filamentous morphology has only been observed in pure culture, and their morphology in activated sludge is unknown (Figure 1, blue). MiDAS phylotypes were linked to previously described phylotypes using the coverage of genus-specific FISH probes (see Table 1). *Ca.* Defluviifilum (morphotype 0803) previously suggested to be implicated in bulking (Kragelund et al., 2011; Speirs et al., 2015), was not included in the analysis as this name is no longer present in MiDAS taxonomy (Nierychlo et al., in press) as the sequences targeted by the FISH probes (Kragelund et al., 2011) span several genus-level phylotypes. It is now partially covered by *Ca*. Amarithrix (Speirs et al., 2019), while other members remain to be characterized.

**TABLE 1:** Summary of filamentous bacteria found in Danish WWTPs with BNR and FISH probes targeting the individual filaments.

Phylum/class	Family	Genus (species)	Morphotype/other names	FISH probes	References
Actinobacteria	Cryptosporangiaceae	*Fodinicola*	–	–	Carlsohn et al., 2008
	Dietziaceae	*Dietzia*	Mycolata	DIE993	Rainey et al., 1995; Muller, 2006
	Intrasporangiaceae	*Tetrasphaera* (species midas_s_328)	*Nostocoida limicola* II	Tetra732	Nguyen et al., 2011; Dueholm et al., 2019
	Microthricaceae	*Candidatus* Microthrix	*M. parvicella, M. calida*	Mpa60	Erhart et al., 1997; Rossetti et al., 2005
	Mycobacteriaceae	*Mycobacterium*	*-*	Myc657	Davenport et al., 2000
	Nocardiaceae	*Gordonia*	Mycolata	Gor596	Lechevalier and Lechevalier, 1974; de los Reyes et al., 1997
	Nocardioidaceae	Nocardioides	–	–	Tóth and Borsodi, 2014
Alphaproteobacteria	Geminicoccaceae	*Candidatus* Alysiosphaera	–	Noli-644	Snaidr et al., 2002; Levantesi et al., 2004
	Rhodospirillaceae	*Defluviicoccus* (species seviourii)	*Nostocoida limicola*, cluster III	DF198	Nittami et al., 2009
	Rhodobacteraceae	*Neomegalonema*	Eikelboom type 021N-like, Meganema	Meg1028 +Meg983	Kragelund et al., 2005, 2006
Bacteroidetes	Saprospiraceae	*Haliscomenobacter*	H. hydrossis	HAL-844	Van Veen et al., 1973; Schauer and Hahn, 2005; Kragelund et al., 2008
Betaproteobacteria	Comamonadaceae	Leptothrix	–	SNA	Wagner et al., 1994a
	Comamonadaceae	*Sphaerotilus*	–	LDI	Wagner et al., 1994a
Chloroflexi	Anaerolinaceae	*Anaerolinea*	–	–	Sekiguchi et al., 2003; Yamada et al., 2006
	Amarolineaceae	*Candidatus* Amarolinea	Eikelboom type 0092	CFX64	Nierychlo et al., 2019
	Anaerolinaceae	*Candidatus* Villigracilis	–	CFX763	Nierychlo et al., 2019
	Roseiflexaceae	Kouleothrix	Eikelboom type 1851	CHL1851	Beer et al., 2002
	Amarolineaceae	*Candidatus* Sarcinithrix	Eikelboom type 0914	CFX67 CFX449/CFX1151	Speirs et al., 2011; Nierychlo et al., 2019
	Ca_Promineofilaceae	*Candidatus* Promineofilum	Eikelboom type 0092 Brachythrix	CFX197	Speirs et al., 2009
	Caldilineaceae	midas_g_105	*Ca.* Amarithrix (type 0675)	CFX194b	Speirs et al., 2017, 2019
	Caldilineaceae	midas_g_344	*Ca.* Catenibacter (type 0041)	CFX86a	Speirs et al., 2017, 2019
	Caldilineaceae	midas_g_1668	*Ca.* Catenibacter (type 0041)	CFX86b	Speirs et al., 2017, 2019
	Ca_Promineofilaceae	midas_g_2111	Filamentous Ardenticatenia	CFX841	Speirs et al., 2015, 2019
Firmicutes	Carnobacteriaceae	*Trichococcus*	*Nostocoida limicola* I	NLIMI91	Liu and Seviour, 2001
	Erysipelotrichaceae	*Turicibacter*	–	–	Bosshard, 2002
	Streptococcaceae	*Lactococcus*	–	Lac93	Nielsen et al., 2012
	Streptococcaceae	Streptococcus	*Nostocoida limicola* I	Strept	Trebesius et al., 2000; Kong et al., 2008
Gammaproteobacteria	Moraxellaceae	Acinetobacter	Eikelboom type 1863 (some)	ACA23A	Wagner et al., 1994b
	Thiotrichaceae	*Beggiatoa*	–	BEG811	Strohl, 2005; Macalady et al., 2006
	Thiotrichaceae	*Thiothrix*	Eikelboom type 021N group I, II, III	G123T	Larkin and Shinabarger, 1983; Howarth et al., 1999; Kanagawa et al., 2000; Nielsen et al., 2000; Aruga et al., 2002; Chernousova et al., 2009
Planctomycetes	Planctomycetaceae	*Candidatus* Nostocoida	*Nostocoida limicola* type III	NLIMIII301	Liu and Seviour, 2001; Liu et al., 2001

**FIGURE 2:**
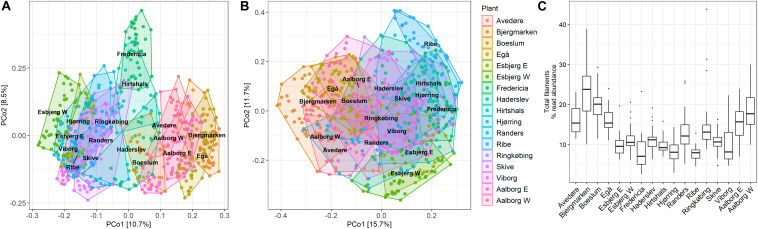
PCoA plot using Bray–Curtis dissimilarity matrix of **(A)** total community; **(B)** filamentous community; and **(C)** boxplot visualizing total filament abundance across the analyzed samples.

The dissimilarities in total community composition in all WWTPs are visualized using PCoA plot (Figure 2A), which shows that samples collected from individual plants over the 13-year period cluster together, indicating a long-term stability of the microbiome. Some plants overlap with one another, suggesting a high degree of similarity in the microbiomes of these plants. Samples from Fredericia WWTP separated the most from other plants, indicating more distinct microbiome composition, presumably due to a high fraction of industrial waste in the influent (Supplementary Table S1). No obvious clustering can be observed in the PCoA plot visualizing the filamentous community (Figure 2B), indicating overall compositional similarities in the filamentous bacteria present in all plants over the years. The separate clustering of some samples from Bjergmarken WWTP is likely caused by very high abundances of *Ca.* Amarolinea in these samples. The filamentous community constituted a significant fraction of all bacteria present (Figure 2C) constituting on average 13.3% of total read abundance, while in some samples exceeding 30% of total read abundance.

Among the bacteria always observed as filaments in activated sludge, the actinobacterial *Ca.* Microthrix was found to be the most abundant genus, with median and mean abundances of 2.3 and 2.9%, respectively, and as high as 16.6% in some samples. The second most abundant filamentous genus was *Trichococcus* (phylum Firmicutes), present at median and average abundances of 2.0 and 2.6%, respectively, and maximum abundance of 12.5%. Other abundant filamentous genera were dominated by bacteria belonging to the phylum Chloroflexi: *Ca.* Villigracilis, with median and mean abundances of 0.7 and 1.0%, respectively, and up to 6.4% in some plants; *Ca.* Promineofilum, with median and mean abundance of 0.5 and 0.9%, respectively, and with maximum of 7% in some samples; and *Ca.* Sarcinithrix, with median and mean abundances of 0.3 and 0.4%, respectively, and present in up to 6.3% of the total reads. Two of the genera with putative filamentous morphology in activated sludge (Figure 1, blue), *Fodinicola* and *Leptothrix*, were present at median abundances of 0.4%, and mean abundances of 0.6 and 0.5%, respectively. Despite the low average abundance values, these bacteria were found to be present in excess of 8 and 4%, respectively, in some of the samples analyzed.

The occurrence of the remaining filamentous bacteria did not exceed the average (and median) abundance of 0.5%. Interestingly, *Ca.* Amarolinea had a median abundance of 0%, and an average abundance of 1.4% but exceeded 33% of the total reads in some plants. Its presence across the plants was noticeably non-uniform, as indicated by the relatively large interquartile range shown in Figure 1. Few of the generally rarely observed filamentous bacteria: *Nocardioides*, *Mycobacterium*, and *Tetrasphaera* species midas_s_328, were found in several samples in abundance exceeding 10% of total reads. Several genera belonging to phylum Chloroflexi: midas_g_105, midas_g_344, midas_g_1668, and midas_g_2111 were present at very low abundances in Danish plants. By analyzing *in silico* the coverage of FISH probes (Speirs et al., 2017, 2019, see Table 1) we identified these as *Ca*. Amarithrix, *Ca*. Catenibacter, and *Ardenticatenia*, respectively.

### Assessment of Activated Sludge Settling Properties

**FIGURE 3:**
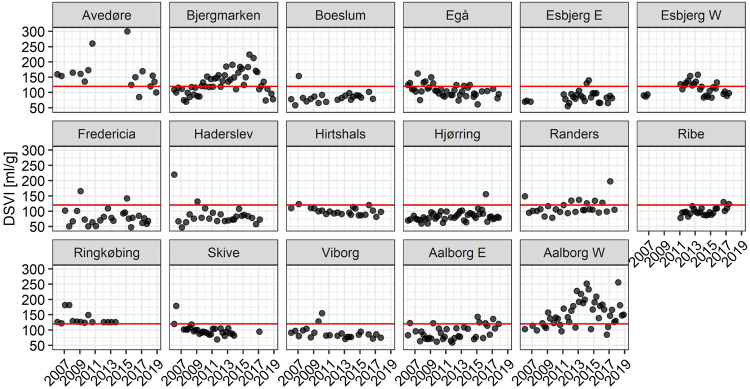
DSVI measurements for the Danish municipal BNR WWTPs for the years 2006–2018. Red line denotes the empirical 120 mL/g upper threshold for sludge with good settling properties.

Activated sludge settling properties were monitored in all plants for 13 years (up to 4 samples per year) with DSVI as an indicator of the biomass sedimentation condition (Figure 3). The majority of the WWTPs had DSVI values below or close to the suggested 120 mL/g threshold for bulking sludge in municipal plants (Mielczarek et al., 2012). Several plants had experienced transient sludge settling problems, as visualized by single elevated DSVI values. Bjergmarken and Aalborg W were identified as WWTPs experiencing long-term issues with biomass settling, with DSVI values above 120 mL/g in both plants measured for several consecutive years, and occasionally reaching values > 200 ml/g. Microscopic analysis of activated sludge samples from these plants, performed over the study period, suggested that observed settleability problems were caused by filamentous bulking (data not shown).

The plants surveyed rarely experienced foaming problems in either the process or settling tanks. This may be due to the routine addition of chemicals [polyaluminum chloride (PAC)] in most plants when foaming problems start. In the few cases recorded, foaming was due to *Ca.* Microthrix, as evaluated by microscopy. *Gordonia* was present in low abundance in a number of plants but was not observed in abundance during foaming episodes.

### Occurrence and Dynamics of Filamentous Bacteria in Bulking Activated Sludge

**FIGURE 4:**
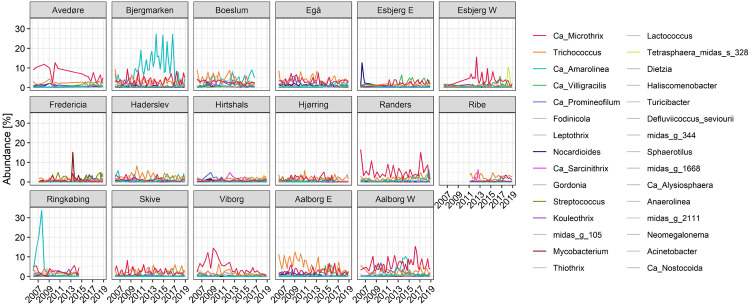
Read abundance of filamentous genera in full-scale Danish WWTPs for the years 2006–2018. The low-abundant filamentous genera are shown in gray, while the abundant ones are shown in color.

The abundance of the known filamentous genera was monitored in all the plants (Figure 4) during 2006–2018. *Ca.* Microthrix and *Trichococcus* dominated in the majority of the plants, and their abundance fluctuated with higher levels of both genera in the cold seasons. Contrary to these, *Ca*. Amarolinea, a recently characterized genus belonging to the phylum Chloroflexi (Andersen et al., 2019; Nierychlo et al., 2019), was a persisting, dominating filament in Bjergmarken WWTP, continuously increasing in abundance in the years 2009–2015, and present in excess of 25% of biomass in some samples. Together with *Ca.* Microthrix, *Ca*. Amarolinea was also a co-dominant filament in Aalborg WWTP, comprising 10–20% of total read abundance in the years 2012–2014. The remaining filamentous organisms were present at a low, stable level in most plants. Transient increases in the abundance of *Nocardioides*, *Tetrasphaera* midas_s_328, and *Mycobacterium* exceeding 10% of total read abundance was observed in Esbjerg E, Esbjerg W, and Fredericia, respectively.

### Relationship Between Filament Abundance and Settling Properties

**FIGURE 5:**
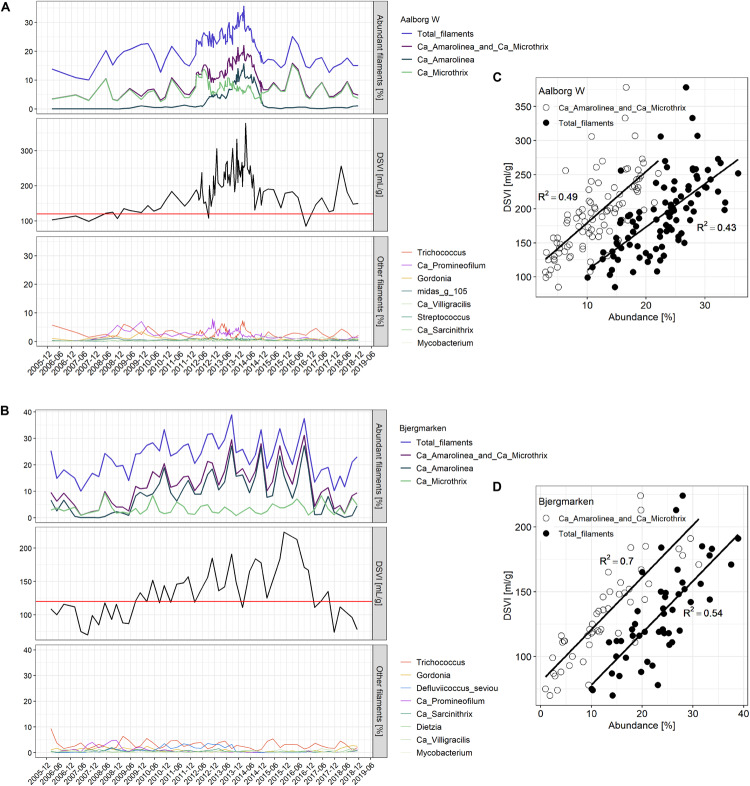
Abundance of filamentous microorganisms and DSVI values for the years 2006–2018 in **(A)** Aalborg West and **(B)** Bjergmarken WWTP; top panel: the abundance of total filaments, *Ca*. Microthrix and *Ca*. Amarolinea and their sum; middle panel: DSVI; bottom panel: the abundance of other filamentous genera in the plant. The relationship between total filaments abundance (solid circles) and summed *Ca.* Microthrix and *Ca.* Amarolinea abundance (empty circles) and DSVI in **(C)** Aalborg West and **(D)** Bjergmarken WWTP (*P*-value for all reported *R*^2^ is < 0.001).

**FIGURE 6:**
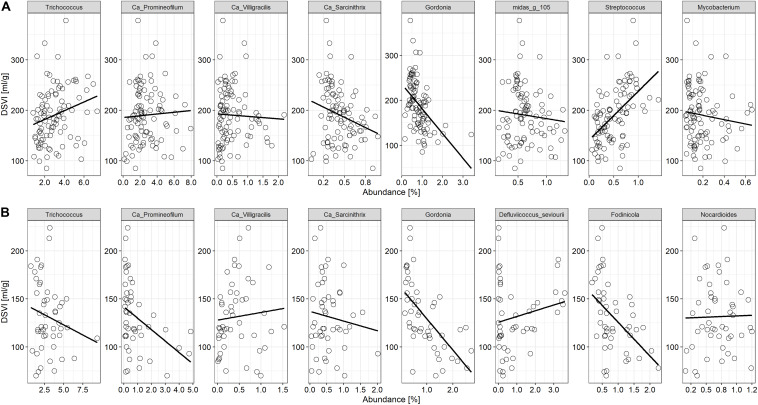
Relationship between the other abundant filamentous bacteria found in activated sludge and DSVI in **(A)** Aalborg W and **(B)** Bjergmarken WWTPs experiencing persistent settling problems.

Based on the analysis of DSVI data, Bjergmarken and Aalborg W were identified as WWTPs experiencing severe sludge settling problems. In order to visualize the link between the filament abundance and the settling properties of the bulking plants, time-series abundances of the filamentous organisms present and measured DSVI values were analyzed (Figure 5). In Aalborg W, the fluctuating abundance of *Ca*. Microthrix coincided with the fluctuation of DSVI values in the years 2006–2011, while the drastic increase of DSVI values to > 200 mL/g was accompanied by the proliferation of *Ca*. Amarolinea. The summed abundance of these two filamentous organisms seemed to explain very well the DSVI pattern observed, while the presence of other filamentous organisms did not seem to follow the DSVI trend (Figure 5A). The linear model of the summed abundance of *Ca.* Amarolinea and *Ca*. Microthrix in Aalborg W and DSVI indicated strong relationship between the occurrence of these two filamentous organisms and the observed settling problems (*R*^2^ = 0.49, Figure 5C). This relationship was stronger than the one found between DSVI and total filament abundance (*R*^2^ = 0.43). Similar to Aalborg W, a strong positive relationship was also found in Bjergmarken between the collective abundance of *Ca*. Microthrix and *Ca*. Amarolinea and the DSVI values (*R*^2^ = 0.7, Figure 5D). Even though *Ca*. Microthrix was present in Bjergmarken at much lower abundance than *Ca.* Amarolinea, it was included in the linear model since it is the most problematic bulking filament in Danish municipal WWTPs. R^2^ value for *Ca*. Amarolinea only vs. DSVI was slightly lower than for the summed abundance of *Ca*. Amarolinea and *Ca.* Microthrix (0.63 vs. 0.70, respectively) demonstrating that in this plant the DSVI was more influenced by the steady increase in abundance of *Ca*. Amarolinea (Figure 5B). *Ca*. Amarolinea was also linked to the transient bulking problems in Ringkøbing WWTP, while *Ca*. Microthrix was associated with elevated DSVI values in Avedøre, Egå, Esbjerg W, and Randers WWTPs (Figures 3, 4). None of the remaining abundant filamentous genera had a clear positive relationship with DSVI in the bulking plants (Figure 6) except *Streptococcus* in Aalborg W, however, it was present at relatively low abundance in the plant.

**FIGURE 7:**
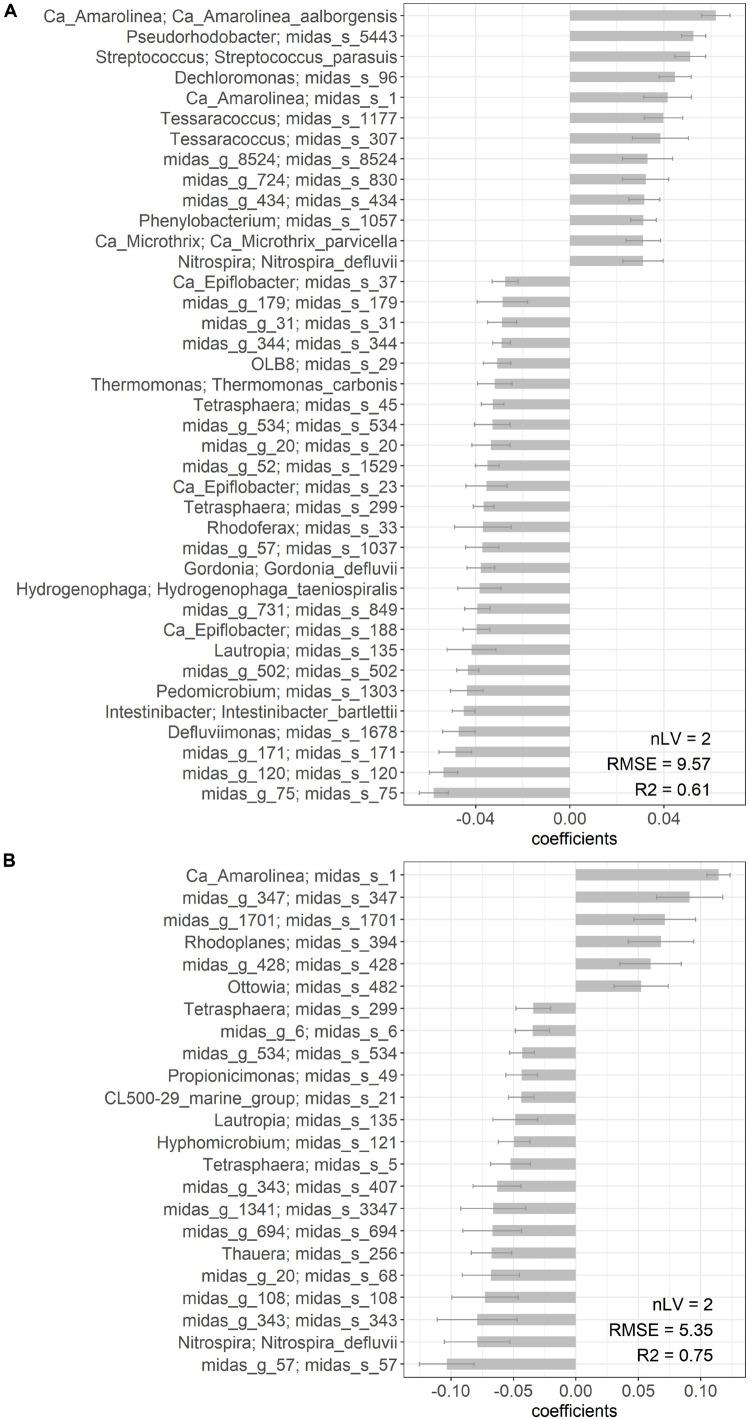
List of species significantly (*P* < 0.05) contributing to the PLS estimation of DSVI parameter in **(A)** Aalborg West and **(B)** Bjergmarken. The number of selected component (latent variables, nLV), root mean square error (RMSE), and *R*^2^ for the cross-validation results are indicated.

PLS regression was applied to predict DSVI using total species-level microbial community composition. Good coefficients of determination were obtained for both models (*R*^2^ = 0.61 for Aalborg W and *R*^2^ = 0.75 for Bjergmarken). The species which significantly (*P* < 0.05) contributed to each PLS model, are shown in Figure 7. Significant contribution of *Ca*. Amarolinea and *Ca*. Microthrix in Aalborg W, and *Ca*. Amarolinea in Bjergmarken to the deterioration of sludge settling properties is indicated by high positive PLS coefficient observed for the species belonging to these two genera (Figure 7). *Ca*. Microthrix was not found to significantly contribute to the PLS model for Bjergmarken, presumably due to the extremely high abundance of *Ca*. Amarolinea over the years, which may mask the contribution of *Ca*. Microthrix to the elevated DSVI values. Other species positively correlated with high DSVI values, mostly belong to the genera without known function in activated sludge. Interestingly, in Aalborg W species belonging to two putative polyphosphate accumulating organisms (PAO) genera, *Dechloromonas* and *Tessaracoccus*, were also shown to negatively influence settling properties, however, the reason or mechanism for this is unclear.

**FIGURE 8:**
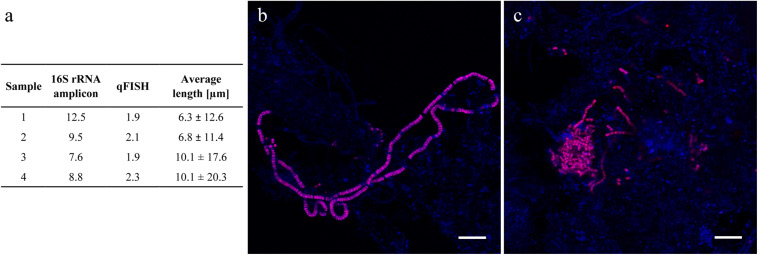
**(a)** Abundance of *Trichococcus* in four Danish activated sludge plants and their corresponding average cell length measurement; **(b,c)** Variable morphology of *Trichococcus* in Danish WWTPs. Scale bar represents 10 μm.

Genus *Trichococcus* was highly abundant in most plants between 2006 and 2018, including the two plants experiencing bulking. Interestingly, its diversity was very low with only one species abundant across all plants (Supplementary Figure S2). Members of the genus are known to grow as both single cells and filaments, so FISH was applied to investigate their morphology in activated sludge. The analyses revealed that *Trichococcus* cells were predominantly present as single cells or short filaments with the average and median length of 8.3 and 1.6 μm, respectively (Figures 8b,c). Furthermore, comparison of qFISH and 16S rRNA amplicon results revealed a substantial difference between its abundance and biovolume, suggesting overestimation of *Trichococcus* by amplicon sequencing (Figure 8a). The average number of 16S rRNA gene copies reported for *Trichococcus* ranges from 1 to 12 (average 5.6),^2^ which is higher than reported for bacteria in general (Angly et al., 2014), supporting the potential overestimation of the abundance of this genus by amplicon sequencing.

### Species-Level Diversity of Bulking Filaments in Full-Scale Danish WWTPs

**FIGURE 9:**
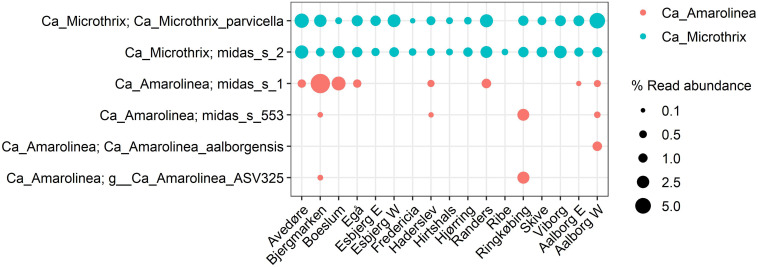
Species-level diversity of *Ca.* Microthrix and *Ca*. Amarolinea based on the 16S rRNA amplicon sequencing. Each data point represents the average abundance in all the samples from given plant included in the analysis.

The species-level diversity of *Ca.* Microthrix and *Ca*. Amarolinea is presented in Figure 9. Both genera were comprised of only few species, indicating low levels of diversity. Only two species were identified for *Ca.* Microthrix and four for *Ca.* Amarolinea. The two *Ca*. Microthrix species co-existed at similar abundances in most plants. Conversely, only one species of the *Ca*. Amarolinea was abundant in any given plant and time, with *Ca*. Amarolinea aalborgensis and *Ca*. Amarolinea midas_s_1 dominating in Aalborg W and Bjergmarken, respectively.

## Discussion

Filamentous bulking is commonly observed in full-scale WWTPs, especially during the winter season (Tandoi et al., 2006). This study revealed the extent of the problem for Danish systems, with 4 of 17 plants experiencing occasional problems with sludge settling, where 2 of these suffered from long-term bulking. The average DSVI in individual plants ranged from 80 to 150 mL/g, however in 27% of the analyzed samples, DSVI was higher than the suggested 120 mL/g threshold for bulking sludge.

The comprehensive survey performed in this study revealed that the collective occurrence of both *Ca.* Amarolinea and *Ca.* Microthrix were responsible for the deteriorated sludge settling in the two Danish plants frequently suffering from severe bulking. These two genera were also linked to transient bulking issues in several other plants. Using a simple linear model, the presence of these two filaments could explain the observed bulking as good as (or better than) when total filamentous community was taken into account. Other filamentous bacteria were not shown to cause serious problems in our study, however, this may be due to the fact that they were not present in high abundances in Danish BNR plants and we cannot rule out their negative impact on settling in general. For example, a number of low-abundance filamentous genera in the Danish WWTPs, such as *Thiothrix*, *Meganema*, *Ca*. Alysiosphaera and others, are primarily known to be problematic in WWTPs treating industrial wastewater as well as in plants without nutrient removal (Van Der Waarde et al., 2002).

**FIGURE 10:**
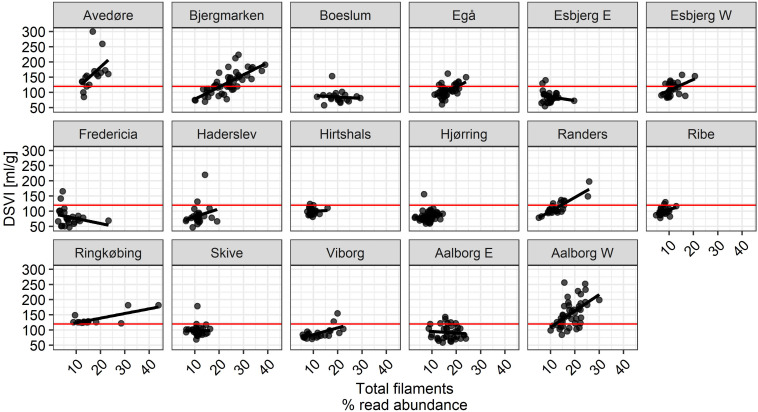
The relationship between total filament read abundance and DSVI. Red line indicates DSVI equal to 120 mL/g SS.

Importantly, it was evident that the total filament abundance alone cannot always explain bulking episodes in BNR plants, as there were some plants with total filament abundances exceeding 20% without visible deterioration of settling properties, while elevated DSVI was observed in other plants with total known filament abundances of only 10–15% (Figure 10). These observations suggest the importance of the type of filament present. Other factors may also contribute to poor settling such as low amount of inorganic particles, presence of certain non-filamentous microorganisms, or presence of filamentous organisms belonging to phylotypes whose identity is unknown. Moreover, it is likely that the combined effect of type, abundance, and localization of the filament affected settling properties of activated sludge flocs.

The link between the proliferation of *Ca.* Microthrix and poor sludge settling properties has been described previously (Rossetti et al., 2005; Guo and Zhang, 2012; Mielczarek et al., 2012), and has been directly linked to increased SVI (Griffin and Wells, 2017). The seasonal variation in abundance observed in this study, with prevalence in cold seasons, is also consistent with other studies (summarized in Rossetti et al., 2005). *Ca.* Microthrix is a specialized lipid consumer shown to excrete lipases (Schade and Lemmer, 2005), take up and accumulate long chain fatty acids under anaerobic conditions, and use it for growth in subsequent aerobic or anoxic conditions (Andreasen and Nielsen, 2000; Hesselsoe et al., 2005). It has the ability to grow with nitrate as electron acceptor, while further reduction of nitrite has not been demonstrated *in situ* (Hesselsoe et al., 2005). *Ca*. Microthrix can also accumulate large amounts of polyphosphate, but its cycling between aerobic and anaerobic conditions, typical for PAO, has not been demonstrated (Andreasen and Nielsen, 2000). Its hydrophobic cell surface (Nielsen et al., 2002) facilitates adsorption of lipids to cell surface, as well as contributes to foaming. Its excessive growth can to some extent be controlled by dosing polyaluminum chloride compounds (Roels et al., 2002), which is a common practice in Danish plants during bulking episodes. The suggested control mechanism includes reduction of lipase activity and decrease of substrate transport inside the cell (Nielsen et al., 2005; Hamit-Eminovski et al., 2010). Conversely, no known control practices exist for *Ca.* Amarolinea. Recent physiological characterization based on its genome (Andersen et al., 2019) and *in situ* studies (Nierychlo et al., 2019) indicate aerobic respiration and fermentation of sugar compounds. The genome of *Ca*. Amarolinea aalborgensis indicates that it may reduce nitrate to ammonium, possibly contributing to the accumulation of the latter inside the WWTPs at the expense of efficient nitrogen removal. *In situ* assessment of their ability to denitrify was inconclusive (Nierychlo et al., 2019) and annotation of the *Ca.* Amarolinea aalborgensis genome did not identify a respiratory nitrite reductase (nirS/nirK, Andersen et al., 2019).

Although *Ca.* Amarolinea has only recently been described, the occurrence of Chloroflexi filaments has long been suggested to have an important role in bulking incidents in activated sludge wastewater treatment plants, particularly those with nutrient removal (Nielsen et al., 2009b; Speirs et al., 2019). Our study confirmed that they constituted a substantial proportion of the activated sludge biomass in most of the full-scale WWTPs surveyed (maximum of > 40% of total read abundance in this study), and their ample presence in many BNR plants has also been confirmed by previous FISH surveys (Bjornsson et al., 2002; Mielczarek et al., 2012; Milobędzka and Muszyński, 2015). The negative effect of Chloroflexi phylotypes on sludge settleability seems to vary, as in this study only *Ca.* Amarolinea was clearly implicated in bulking. Different Chloroflexi phylotypes have different localization in the floc skeleton, which has important implications for their perceived role in filamentous bulking. *Ca.* Sarcinathrix (as morphotype 0914) as well as *Ca*. Villigracilis are largely located inside the floc (Speirs et al., 2011; Nierychlo et al., 2019), while *Ca.* Amarolinea and *Ca.* Promineofilum protrude from the floc where they are suggested to contribute to interfloc bridging in bulking sludge (Speirs et al., 2009; Nierychlo et al., 2019). In our study, only the *Ca.* Amarolinea seemed to influence sludge settling. The results demonstrate that visualization with FISH may be important to evaluate filament localization and potential effect on floc structure, but cannot always predict their effect on settling properties as evaluated by DSVI.

Additionally, the unknown effect of sub-genus diversity may be important for the sludge settling properties. Contrary to popular public taxonomic databases, the use of the ecosystem-specific MiDAS 3 database provides taxonomic annotation to the sub-genus level allowing population dynamics to be studied at the species level (Nierychlo et al., in press). The value of such an approach is demonstrated in this study where species of the abundant *Ca.* Amarolinea were shown to differentially proliferate in Danish plants, indicating potentially different physiologies that may require development of species-specific control measures. Similar observations were made for *Ca*. Microthrix and other filamentous genera, each comprising from 1 to 5 species (Figure 9 and Supplementary Figure S2). Since no information about individual species belonging to the filamentous genera is available, further studies will help to discriminate potential physiological or morphological differences among them. A prominent example of the value of the species level resolution provided by MiDAS is in the ability to link the species-level-phylotype *Tetrasphaera* midas_s_328 with a filamentous morphology via the design and application of FISH probes, whereas the rest of the genus is non-filamentous (Dueholm et al., 2019).

The high abundance of genus *Trichococcus* observed in the current study has also been observed previously (Guo and Zhang, 2012; Wang et al., 2016). A high abundance in influent wastewater indicates that filaments from this genus may be continuously seeded from the sewage, and also appear to grow within the BNR systems (VandeWalle et al., 2012; Saunders et al., 2016; Kristensen et al., 2020). However, the current study suggests that the abundance and subsequent importance of the genus is overestimated by amplicon sequencing. These filaments have previously been identified as morphotype *Nostocoida limicola* I by light microscopy, and suggested to be implicated in bulking (Liu and Seviour, 2001; Wang et al., 2016), however, its low biovolume and rod-shape rather than filamentous form prevailing in activated sludge, as shown in this study, indicate that its negative influence on sludge settling is not likely.

Bacteria belonging to the phylum Saccharibacteria (formerly known as TM7) were previously shown to include filamentous phylotypes (Hugenholtz et al., 2001; Kindaichi et al., 2016). *In silico* analysis of the FISH probe coverage using MiDAS3 database revealed that probes TM7305 and TM7905 (Hugenholtz et al., 2001) target a number of different families in the order Saccharomonadales, while probes Sacch720a, Sacch720b, and Sacch933 (Kindaichi et al., 2016) target multiple genera across a number of different families in same order. Therefore, it was not possible to infer potential filamentous morphology for individual genera in the phylum Saccharibacteria in this study.

All bacteria found in activated sludge community for which filamentous morphology has been observed *in situ* or in pure culture studies were included in this survey. We believe that the majority of filamentous microorganisms that are abundant in Danish plants has already been identified, however, since the list of the abundant bacteria in activated sludge ecosystem contains unknown genera (Nierychlo et al., in press), we cannot exclude that some of these possess filamentous morphology. Our ongoing study of bacteria from phylum Chloroflexi (unpublished data), possessing many filamentous representatives in activated sludge, revealed several unknown genera that are occasionally abundant in Danish WWTPs.

Additionally, the nature of amplicon sequencing itself may introduce a bias in the observed filamentous community composition. Even though primers targeting V1-3 region of 16S rRNA are generally recommended for the analysis of the activated sludge (Albertsen et al., 2015; Dueholm et al., 2019), some filamentous taxa may not be targeted efficiently during the PCR amplification step. Such example may be the genus *Gordonia*, which in this study was detected in low abundance in Danish plants. Evaluation of the V1-3 primer performance for *Gordonia* sequences present in MiDAS3 database revealed several mismatches to the forward primer, potentially leading to underestimation of *Gordonia* abundance.

## Conclusion

In this study we have performed a comprehensive survey of filamentous bacteria present in municipal BNR Danish WWTPs. Many genera were found but only two in an abundance that caused serious bulking problems: the well-known *Ca.* Microthrix and the recently characterized genus *Ca.* Amarolinea (phylum Chloroflexi). These two were responsible for the majority of severe bulking episodes in municipal Danish WWTPs observed over the 13-year sampling period. Notably, it is the composition, and not just the collective abundance, of the filamentous community that is important for the deterioration of sludge settling properties. Different species of *Ca*. Amarolinea were dominating in the plants with bulking problems, which indicates their adaptation to different niches and potentially complicates the development of control methods. Other important, yet unknown, filamentous genera may still be present in activated sludge, thus consistent identification of all the abundant phylotypes is an important future task. This should be followed by the species-level physiological characterization of these filamentous phylotypes to facilitate formulation of targeted control strategies for unwanted filament growth.

## Data Availability Statement

The datasets generated for this study can be found in the Sequence Read Archive (https://www.ncbi.nlm.nih.gov/sra) PRJNA622675, Sequence Read Archive (https://www.ncbi.nlm.nih.gov/sra) PRJNA625645.

## Author Contributions

SM and PN devised the study and its main conceptual ideas with contributions from MN. MN and AZ performed data analysis with contributions from MS-B. CJ and SK performed PLS. ZK performed FISH analyses. The manuscript was drafted by MN and AZ and revised by SM and PN.

## Conflict of Interest

The authors declare that the research was conducted in the absence of any commercial or financial relationships that could be construed as a potential conflict of interest.

## Footnotes

1 https://www.ncbi.nlm.nih.gov/sra

2 https://img.jgi.doe.gov/
