# Supplementary material for: Candidatus Amarolinea and Candidatus Microthrix Are Mainly Responsible for Filamentous Bulking in Danish Municipal Wastewater Treatment Plants
Source: Front Microbiol. 2020 Jun 9;11:1214. doi: 10.3389/fmicb.2020.01214 (PMC7296077; doi:10.3389/fmicb.2020.01214)
Supplement: Supplementary file 1 [file Data_Sheet_1.PDF]

Table S1. Design and sample information for the WWTPs included in the study.

**WWTP**	**Configuration**	**Design**	**Industrial waste [% COD]**	**Primary settling**	**Digester**	**No. of samples analyzed**
Avedøre	Alternating	EBPR	25	Yes	Yes	18
Bjergmarken	Alternating	EBPR	20	No	Yes	52
Boeslum	Recirculation	EBPR	5	No	No	24
Egå	Recirculation	EBPR	40	No	No	54
Esbjerg E	Recirculation	BNR	60	Yes	Yes	36
Esbjerg W	Recirculation	BNR	60	Yes	Yes	35
Fredericia	Recirculation	EBPR	75	No	Yes	37
Haderslev	Alternating	EBPR	5	No	No	33
Hirtshals	Alternating	EBPR	60-70	No	No	26
Hjørring	Recirculation	EBPR	30	Yes	Yes	49
Randers	Recirculation	EBPR	5	Yes	Yes	33
Ribe	Recirculation	EBPR	20	No	No	32
Ringkøbing	Alternating	EBPR	10	Yes	Yes	19
Skive	Recirculation	EBPR	20-65	No	No	49
Viborg	Recirculation	BNR -> EBPR	10	Yes	Yes	36
Aalborg E	Alternating	EBPR	10	No	Yes	49
Aalborg W	Alternating	EBPR	30	Yes	Yes	44
