# Supplementary material for: Candidatus Amarolinea and Candidatus Microthrix Are Mainly Responsible for Filamentous Bulking in Danish Municipal Wastewater Treatment Plants
Source: Front Microbiol. 2020 Jun 9;11:1214. doi: 10.3389/fmicb.2020.01214 (PMC7296077; doi:10.3389/fmicb.2020.01214)
Supplement: Supplementary file 2 [file Data_Sheet_2.PDF]

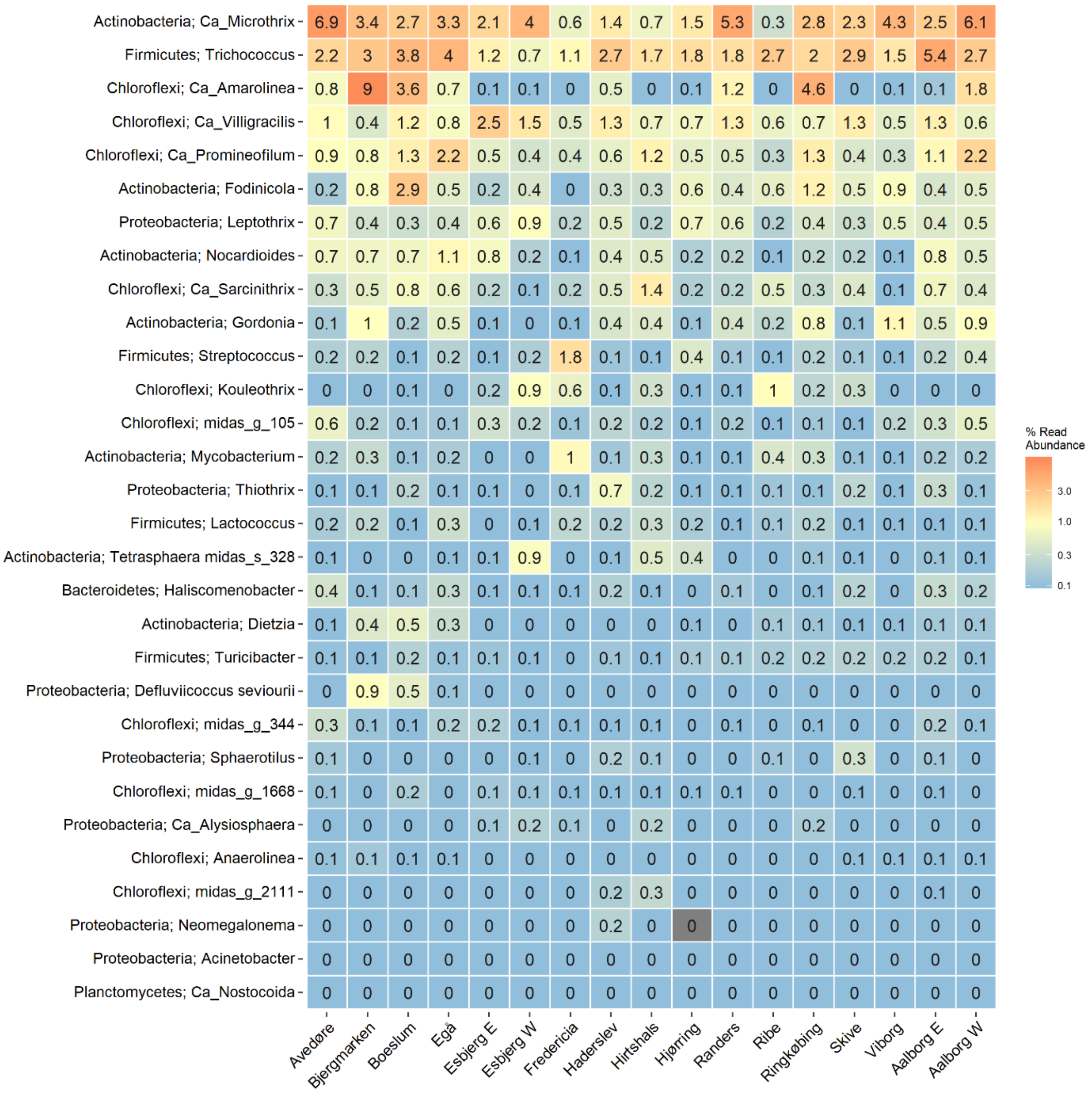

Figure S1. Heatmap showing mean read abundance of filamentous bacteria in percent of all amplicon reads. The numbers represent the average read abundance for the given WWTP. The survey was performed in the years 2006–2018 with up to four samples per year for each plant. Phylum and genus taxonomic information is shown.
