# Supplementary material for: Candidatus Amarolinea and Candidatus Microthrix Are Mainly Responsible for Filamentous Bulking in Danish Municipal Wastewater Treatment Plants
Source: Front Microbiol. 2020 Jun 9;11:1214. doi: 10.3389/fmicb.2020.01214 (PMC7296077; doi:10.3389/fmicb.2020.01214)
Supplement: Supplementary file 3 [file Data_Sheet_3.PDF]

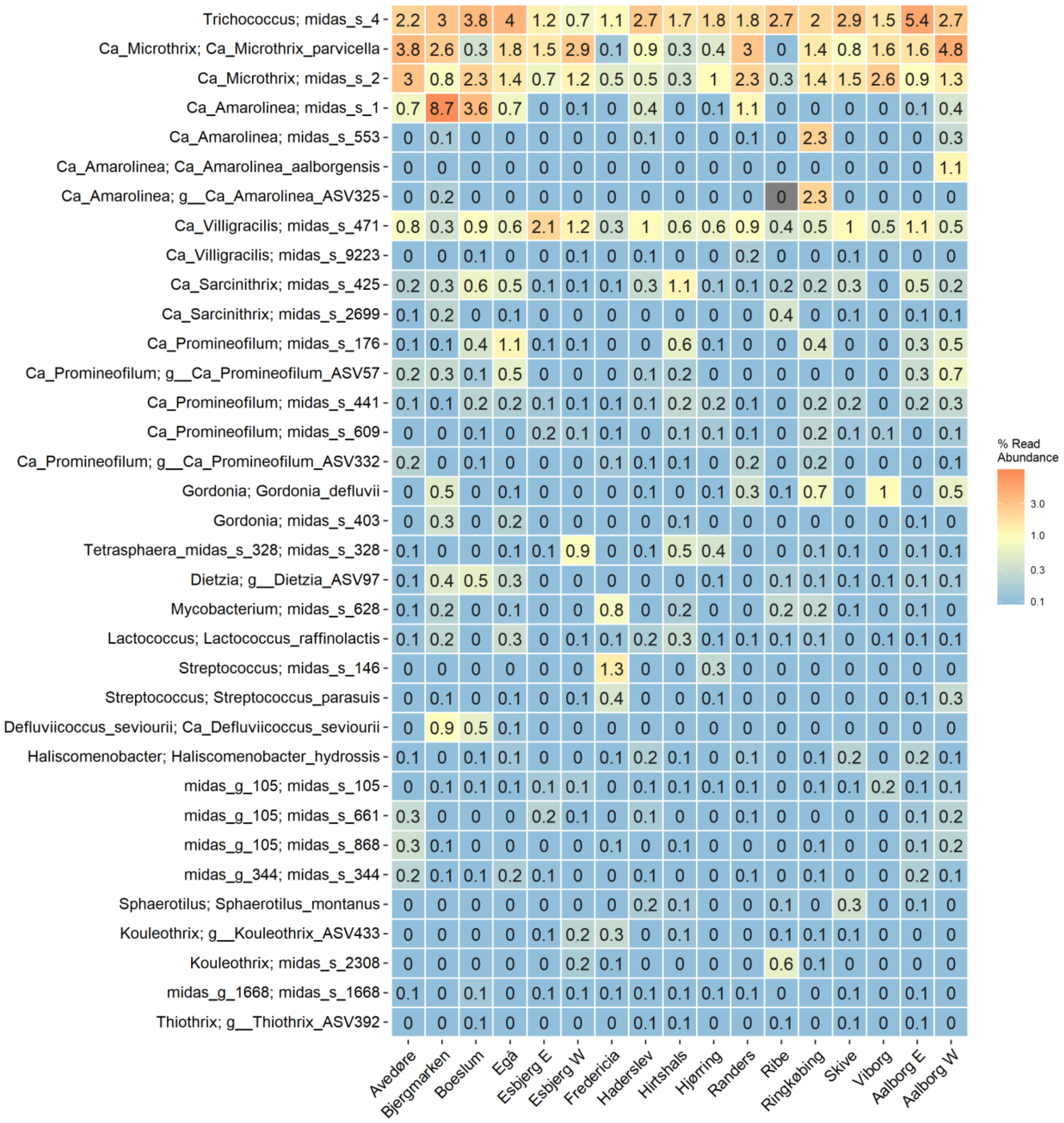

Figure S2. Species-level diversity of the filaments found in Danish WWTPs. Species occurring at mean abundance >= 0.1% are shown. Each number represents the average abundance in all the samples from given plant included in the analysis.
